# Exploring the effect of the primary care health workers number on infectious diarrhea morbidity and where the health resources should go

**DOI:** 10.1038/s41598-022-10060-y

**Published:** 2022-04-11

**Authors:** Xujing Guan, Tianjiao Lan, Weibin Liao, Xue’er Wu, Jay Pan

**Affiliations:** 1 HEOA Group, West China School of Public Health and West China Fourth Hospital, Sichuan University, Chengdu, 610041 China; 2 Sichuan Center for Disease Control and Prevention, No.6, Middle School Road, Chengdu, 610041 China; 3 Institute for Healthy Cities and West China Research Center for Rural Health Development, Sichuan University, Chengdu, 610041 China

**Keywords:** Health policy, Health care economics

## Abstract

We aimed to explore the association between the number of primary healthcare workers and infectious diarrhea morbidity at community levels and to provide evidence-based implications for optimizing primary healthcare manpower resource allocations. We collected annual infectious diarrhea morbidity and relevant data of 4321 communities in Sichuan Province, China, from 2017 to 2019. Global and local Moran’s I were calculated to detect the spatial clustering of infectious diarrhea morbidity and to identify areas where increased primary healthcare manpower resources should be allocated. The spatial lag fixed effects panel data model was adopted to explore the association between the number of primary healthcare workers per 1000 residents and infectious diarrhea morbidity. Significantly high–high and low–low clusters of infectious diarrhea cases were found to be mainly distributed in underdeveloped and developed areas during the studied period years, respectively. The infectious diarrhea morbidity was found to be statistically negatively associated with the number of primary healthcare workers per 1000 residents with a coefficient of − 0.172, indicating that a 0.172 reduction of infectious diarrhea morbidity (1/10,000) was associated with doubled amounts of primary healthcare workers per 1000 residents. Our findings highlighted the role of primary healthcare in the process of infectious diarrhea prevention and control, and implied that constant efforts should be addressed to facilitate infectious diarrhea prevention and control, especially in the underdeveloped areas.

## Introduction

Diarrhea remains a major threat to public health around the world. According to the Global Burden of Disease^1^, in 2016, diarrhea ranked as the eighth leading cause of death among all age groups and the fifth leading cause of death for children less than five years old. Residents mostly affected by diarrhea as a major threat to populational health, especially for children aged less than five years old were found to be mainly distributed in underdeveloped regions including Africa, South East Asia and the Eastern Mediterranean^2,3^. Under-five children in developing countries suffered from an average of 2.9 diarrhea onsets each year^3^, with approximately one-third of total onsets being moderate or severe cases^4^. In China, more than a million diarrhea cases were reported in 2018 alone, with the morbidity of diarrhea found to be 92 cases per hundred thousand population, making it the second highest among notifiable diseases in terms of incidence^5^.

Infectious diarrhea, a gastrointestinal infection, can be caused by a wide range of pathogens, including bacteria, viruses, and parasites^6^. A critical transmission route of infectious diarrhea is the fecal–oral route, such as the consumption of fecally contaminated food, drinking water as well as via person-to-person contact due to poor hygiene^7–9^. The morbidity and distribution of infectious diarrhea would be affected by various factors, including sociodemographic factors (age, education, income etc.)^10–12^, environmental and sanitation factors (poor access to a good water source and poor sanitation)^13,14^, and climate factors (rainfall, temperature and humidity)^15–17^.

Diverse efforts have been made in attempt to reduce the morbidity of infectious diarrhea in a worldwide range, among which improved water, sanitation, and hygiene (WASH) facilities such as piped water, protected shallow wells, and non-shared toilets have been widely accepted as cost-effective ways for reducing the morbidity of infectious diarrhea^18–20^. In China, the rapid socioeconomic development has significantly improved the nationwide penetration of improved water, sanitation, and hygiene facilities. For example, according to National Statistical Yearbook, in urban regions, the penetration of piped water increased from 63.9% in 2000 to 98.36% in 2018^21^. However, various factors have posed huge obstacles for Chinese residents in obtaining actual access to improved WASH^22^. For example, in 2008, 2.81 million disability-adjusted life years (DALYs) and 62,800 deaths were attributed to unsafe water and poor sanitation or hygiene in China. Water pollution in the nation was found to be inadequately controlled^23^ as 44% of nationwide rural water supplies failed to meet minimum drinking water quality standards^24^. Under such circumstances, it is noteworthy that improved WASH facilities are not necessarily associated with improved hygiene behaviors such as safe feces disposal or improved handwashing procedures, which has been highlighted by researchers such as Lamichhane^25^ while has received relatively inadequate investigation based on previous literature.

As improved hygiene behaviors should be addressed as an indispensable aspect in achieving improved public health outcomes in addition to the enhancement of WASH facilities, the optimization of health education programs as well as health service delivery at primary healthcare level should be emphasized as an essential strategy in improving residents’ actual access to improved WASH thus further reducing the incidence of infectious diarrhea. Specifically, primary healthcare institutions have been playing critical roles in China as gatekeepers throughout the process of infectious disease prevention and control, including cutting off transmission routes, protecting vulnerable populations, providing treatment for infected patients as well as providing health educational programs and assisting in the maintenance of WASH facilities among communities. Under such context, it is not difficult to imagine that improved WASH maintenance and health education programs at primary healthcare level are very much likely to achieve the reduction of nationwide infectious diarrhea morbidity via improving residents’ appropriate use of WASH facilities, minimizing the infection of well water in rural areas via disinfection procedures as well as monitoring the concentration of bacterial accumulation in water pipes on a regular surveillance basis.

Despite the significant contributions that primary healthcare institutions have been playing in the process of infectious diseases prevention and control, studies focused on investigating the roles of primary healthcare workers in reducing infectious diarrhea morbidity remain limited based on previous literature. Specifically, a couple of studies^26–28^ have verified the effectiveness of regular visits conducted by community healthcare workers (similar with primary healthcare workers) in reducing childhood infectious diarrhea morbidity, while several other studies^29–32^ have highlighted the significant role of community healthcare workers in improving residents’ health literacy. However, evidences collected under the context of China’s healthcare system were found to be very limited in this aspect, while none of the currently existed studies have ever been focused on evaluating healthcare resource allocation as a determinant for healthcare institutions’ performances in reducing residents’ infectious diarrhea morbidity, especially at primary healthcare level where the distribution of health resource is mainly reflected by the number of healthcare professionals.

In order to bridge such gap embedded in previous literature, this study has been designed for exploring the association between the number of primary healthcare workers per 1000 residents and residents’ infectious diarrhea morbidity in the community range in order to provide evidence-based suggestions for healthcare manpower resource allocation at the community level. In addition, our findings were expected to provide practical implications for other infectious diseases as infectious diarrhea has a list of characteristics typical of communicable diseases including high incidence, diverse pathogens and transmission routes, as well as would be affected by sociodemographic factors and the construction of health infrastructures at regional levels. Infectious diarrhea morbidity and relevant data from 2017 to 2019 in Sichuan Province, China were collected for analysis. The spatial lag fixed effects panel data model was adopted to explore the relationship between the number of primary healthcare workers per 1000 residents and infectious diarrhea morbidity in the community range. The local indicators of spatial association (LISA, Local Moran’ *I*) analysis was used to determine areas where increased healthcare manpower resources should be allocated.

This study was expected to contribute to the relevant literature in two aspects. First, our study was expected to bridge the gap embedded in similar studies through exploring the association between the number of primary healthcare workers and infectious diarrhea morbidity at community levels. Through identifying the role of primary healthcare manpower resource allocation in infectious diseases prevention and control in China, our findings were also expected to assist in the formulation of region-specific policies by providing evidences on specific locations of high incidence clusters. Second, our study was expected to add evidences to previously published studies in this field which were conducted at county levels via providing new evidences collected at community levels, which served as a better solution to heterogeneity issue as the spatial variation of disease morbidity would be detected and analyzed at a smaller health administrative unit.

This paper has been structured to contain the following sections. Specifically, an overview of health authorities as well as their roles in the process of infectious diseases prevention and control was briefly described in the “Background” section. The study area, data sources and empirical strategies were described in the “Methods” section, which was followed by “Results” and “Discussions” sections where findings and discussions were provided, respectively.

## Background

In China, at least 23 departments are involved in the process of infectious diseases prevention and control, which can be divided into 6 categories based on their functionalities, including governments, Health Commissions, three kinds of specialist institutions, four kinds of social insurance institutions, thirteen kinds of supportive departments, and other organizations^33–35^.

Specifically, governments are responsible for providing surveillance on infectious diseases prevention and control from a holistic perspective via the initiation and management of projects proposed for communicable disease prevention and control. Specific tasks related to achieving project goals are planned and managed by Health Commissions, for which social insurance institutions such as health insurance institutions will be responsible for providing support in multiple aspects including funding, human resource and substances needed for disease prevention and control^35^. Other supportive departments and organizations in various industries such as education, academic research, technology and industry will also assist in achieving project goals and deliverables within their own scope of responsibilities. However, it is three kinds of specialist institutions that have been playing pivotal roles throughout the process of infectious disease prevention and control, namely specialized public health institutions (such as Center for Disease Control and Prevention), medical institutions and primary healthcare institutions. Specifically, technical and surveillance plans in response to disease outbreaks as well as for disease prevention and control will be proposed and managed by specialized public health institutions throughout both project development and implementation stages^36^. Medical institutions are responsible for providing treatment for patients infected by communicable diseases as well as reporting cases identified^35^, while primary healthcare institutions serve as the gatekeepers for communicable disease prevention and control at community levels, which typically include community health service centers, township health centers, and village clinics in China where all kinds of projects and plans proposed at health administrative levels will be actually implemented^36^. Tasks expected to be accomplished at primary healthcare levels can be summarized as three aspects. First, controlling sources of infection via monitoring, reporting, and treating patients infected by communicable diseases. Second, cutting off all transmission routes via multiple methods such as water, soil, air sampling and testing, monitoring accumulated bacterial concentration in water pipes, mosquitoes and flies control, sterilization, and disinfection. Third, protecting vulnerable populations via the provision of health education programs for residents in order to improve their knowledge about appropriate hand washing behaviors, safe water consumption tips, and appropriate use of hygiene facilities.

At present, the lack of healthcare workers in primary healthcare institutions remains a critical problem in China. As the consequence, projects proposed for infectious disease prevention and control have been difficult to be implemented or to achieve expected goals. In Sichuan Province, there were only 22.47 primary healthcare workers per ten thousand population and only 2.34 health workers per primary healthcare facility in 2019^37^. It was reported by governmental officers from Sichuan Health Commission who had been in charge of communicable disease prevention and control that the lack of manpower resources as well as inadequate knowledge of communicable disease among primary healthcare workers have posed huge obstacles for primary healthcare providers in achieving desired quality and efficiency of healthcare delivery in spite of their full engagement in most of the infectious disease cases identified. Taking health education as an example, more than 90 percentage of the workers engaged in health education were found to be part-time or even multi-tasking^38–40^, thus resulting in 45 percentage of primary healthcare facilities incapable of providing effective health education programs^40,41^. Among primary healthcare institutions where services have been actually provided for health education purposes, residents engaged in health education programs only accounted for 60% of the targeted populations^42,43^.

## Results

**Figure 1:**
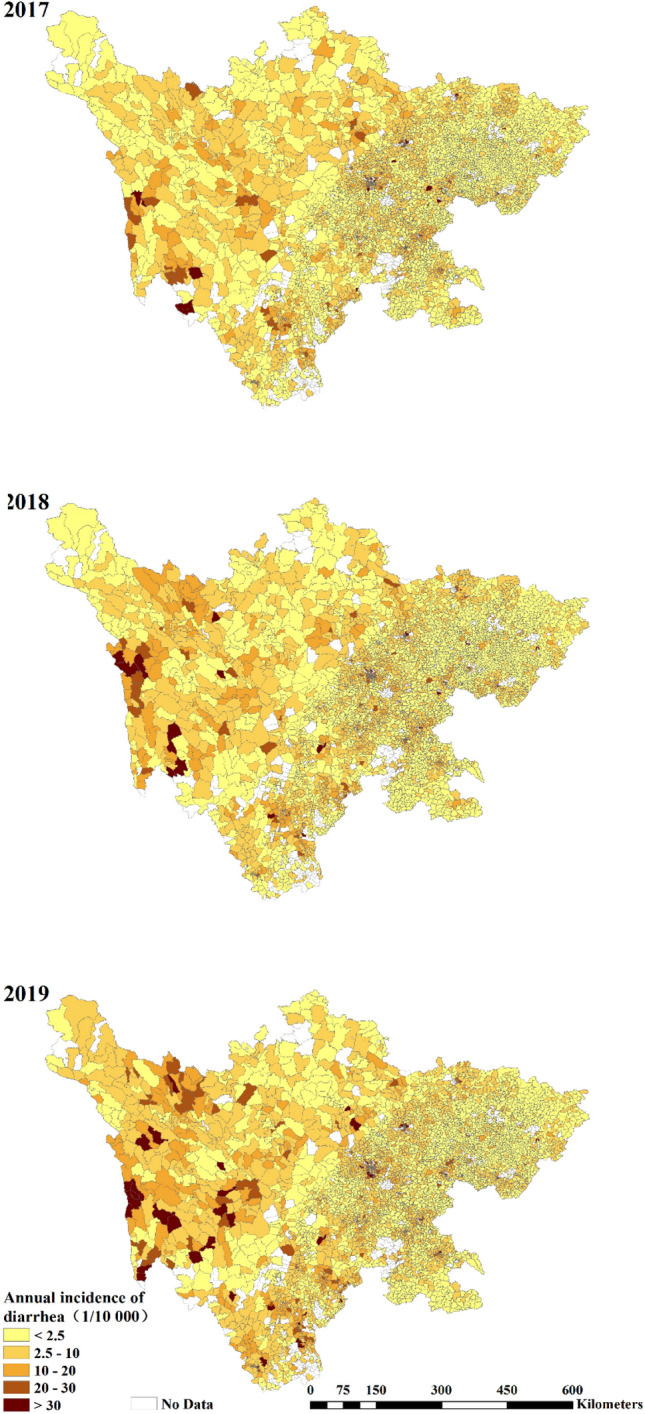
The geographical distribution of infectious diarrhea morbidity at community-level from 2017 to 2019. The map was created by ArcGIS software (version 10.0, authorization number: EFL734321752, URL: https://developers.arcgis.com/).

Annually, the infectious diarrhea morbidities in Sichuan Province were found to be 4.20, 4.36, and 4.62 per 10,000 people from 2017 to 2019. As shown in Fig. 1, the high morbidity clustered areas were mainly distributed in western Sichuan, while low morbidity clustered areas were mainly distributed in the middle and eastern regions in Sichuan where the economic development status and primary healthcare resource allocations were relatively superior than those in western Sichuan. It can be seen that this kind of trend had become more pronounced during the changing period of time, with the constant emergence of high infectious diarrhea morbidity communities in western Sichuan.

Infectious diarrhea morbidities at the community level in Sichuan Province from 2017 to 2019 were descripted in Table 1, with the average morbidity of each year found to be 3.5, 3.8, and 4.4, and the maximum morbidity of each year found to be 53.0, 71.9, and 149.1, respectively. In addition to infectious diarrhea morbidity, the number of primary healthcare workers and subsidy per staff also demonstrated significant variation across communities in Sichuan Province.

**Table 1:** Descriptive statistics of key variables at community-level in Sichuan Province, China, from 2017 to 2019.

Variables	2017			2018			2019		
	Mean ± SD	Maximum	Minimum	Mean ± SD	Maximum	Minimum	Mean ± SD	Maximum	Minimum
Morbidity (1/10,000)	3.5 ± 5.3	53.0	0	3.8 ± 5.9	71.9	0	4.4 ± 6.8	149.1	0
Number of primary care health workers	23.9 ± 28.5	415.0	1.0	24.9 ± 29.4	460.0	1.0	25.9 ± 30.5	481.0	1.0
Subsidy per staff	103.4 ± 69.8	1673.8	0	61.7 ± 105.8	976.6	0	103.7 ± 57.9	856.2	0
Population	153.3 ± 161.8	2675.0	6.1	153.7 ± 162.7	2689.2	6.0	154.1 ± 163.7	2724.8	6.1

The unit of subsidy per staff is thousand Yuan; the unit of population is a hundred person.

### Spatial autocorrelation analysis of infectious diarrhea morbidity

**Figure 2:**
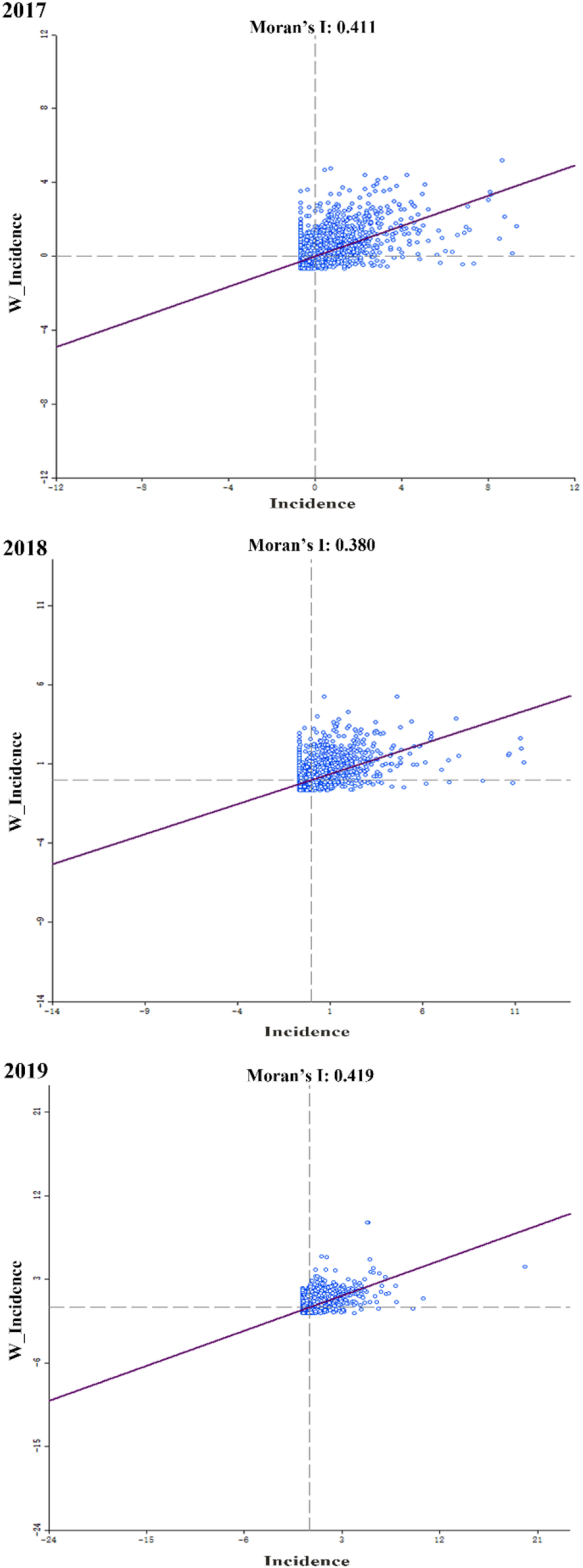
Moran scatter plot for the annual infectious diarrhea morbidity at community-level from 2017 to 2019.

**Figure 3:**
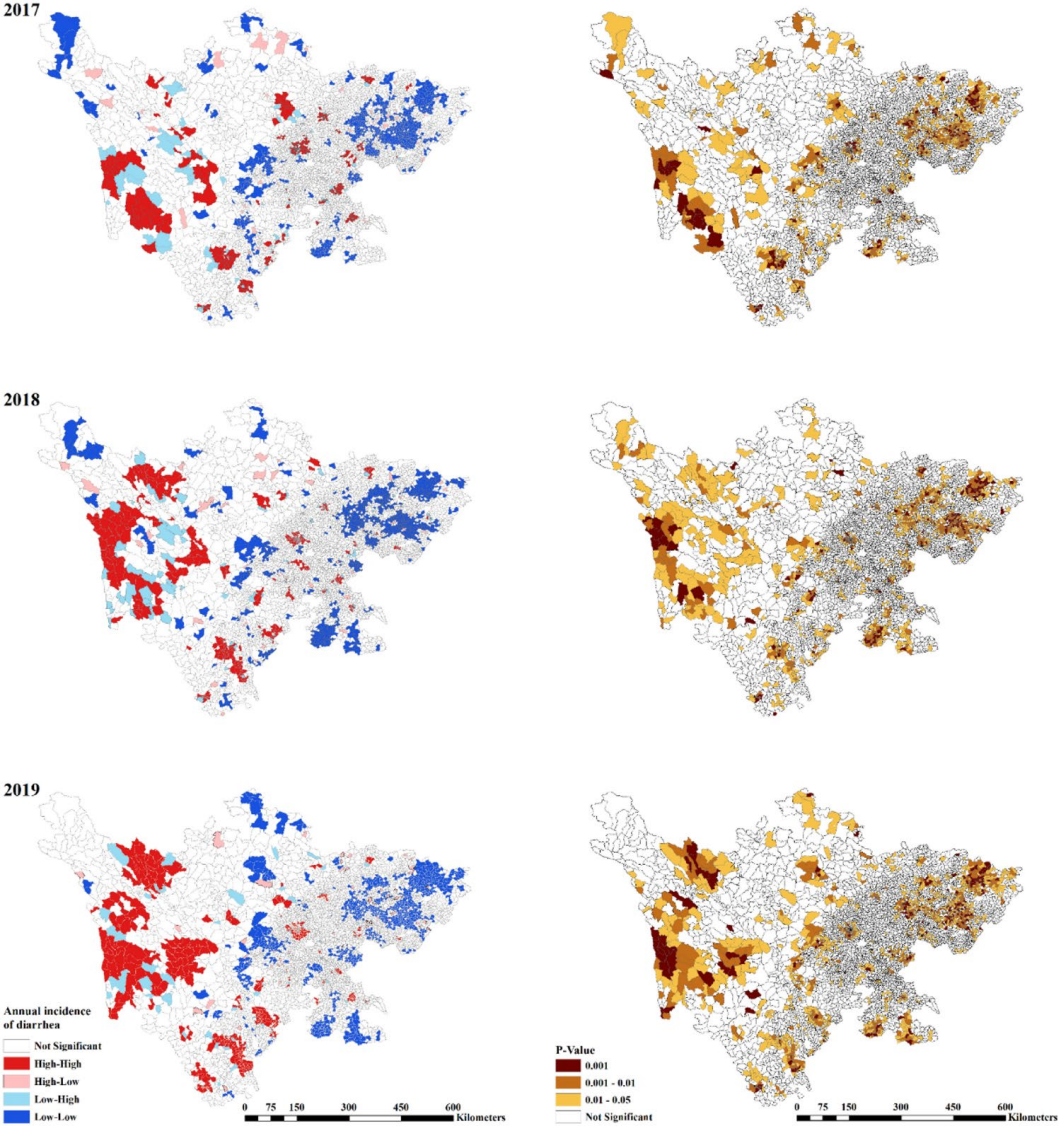
LISA significance map and cluster map for infectious diarrhea morbidity at community-level in Sichuan Province, China, 2017–2019. The map was created by ArcGIS software (version 10.0, authorization number: EFL734321752, URL: https://developers.arcgis.com/).

As shown in Fig. 2, the values of global Moran’s I between 2017 and 2019 at community-level were high, ranging from 0.38 to 0.42 with all *p* values < 0.01. The results identify the positive global autocorrelation of infectious diarrhea morbidity, namely the communities with high infectious diarrhea morbidity tend to be adjacent to the communities with high morbidity, and vice versa. LISA analysis revealed four types of spatial clusters in terms communities. As shown in Fig. 3, the significantly high–high and low–low clusters were mainly concentrated in western and eastern Sichuan across years, which corresponds to the distribution of the regional economic development and the primary healthcare resources (eastern Sichuan possess better primary healthcare resources while western Sichuan is in the opposite situation). Besides, this trend tended to be pronounced as the time went on, with more high–high clusters appear in western Sichuan.

### Spatial panel analysis

We initially conducted several model specification tests to obtain the appropriate model. Hausman test robust to spatial autocorrelation was firstly employed to determine whether a fixed effect or a random effect model should be chosen. As a result, a significant fixed effect of each community was found. Subsequently, based on a fixed effect model, we conducted Lagrange multiplier tests to identify whether a spatial lag effect or a spatial error effect model should be selected. The results showed that the spatial lag effect was more significant than the spatial error effect. Therefore, we finally used the spatial lag fixed effects panel data model to examine the associations between infectious diarrhea morbidity and the number of primary healthcare workers per 1000 residents (Table 2).

**Table 2:** Results of model specification.

Tests	Test statistics	df	*p* value
Hausman test robust to spatial autocorrelation	65.78	3	< 0.001
LM test for spatial lag dependence	1830.48	1	< 0.001
LM test for spatial error dependence	1825.80	1	< 0.001
Locally robust LM test for spatial lag dependence sub spatial error	1.870	1	0.171
Locally robust LM test for spatial error dependence sub spatial lag	0.834	1	0.361

(1) The number of healthcare workers per 1000 residents, subsidy per staff, and GDP per capita were included as covariates in the test models. (2) Lagrange multiplier (LM) tests were conducted based on fixed effect models.

Based on Eq. (1), the empirical model was fitted at the community-level. The results in Table 3 show that after controlling for subsidy per staff and GDP per capita, the relationship between number of primary healthcare workers per 1000 residents and the infectious diarrhea morbidity was significantly negative. The coefficient of the number of primary care health workers was − 0.172, indicating the infectious diarrhea morbidity (1/10,000) would decrease 0.172 when doubling the number of primary care health workers. Besides, the coefficient of subsidy per staff was − 0.304, representing the infectious diarrhea morbidity (1/10,000) would decrease 0.304 when doubling the subsidy per staff. The spatial autocorrelation coefficient was 0.435 and statistically significant, meaning that a spatial spillover phenomenon existed.

**Table 3:** Results of spatial lag fixed effects panel data model for the infectious diarrhea morbidity (1/10,000).

Variables	(1)	(2)	(3)
Number of healthcare workers per 1000 residents	− 0.151* (0.080)		− 0.172** (0.081)
Subsidy per staff		− 0.227* (0.134)	− 0.304** (0.015)
GDP per capita	1.194*** (0.303)	1.186*** (0.303)	1.184*** (0.303)
Year	Yes	Yes	Yes
*ρ*	0.435*** (0.011)	0.435*** (0.011)	0.435*** (0.011)
*N*	12,963	12,963	12,963

(1) Number of healthcare workers, subsidy per staff, and population are natural log transformed in regression analysis; there are 18 observations of subsidy per staff equaling to 0 and thus transformed to 1 before nature log transformed. (2) GDP per capita represents county economic development. (3) The unit of subsidy per staff and population are 1000 Yuan and 10,000 person, respectively. (4) *ρ* represents spatial autocorrelation coefficient. (5) Standard errors are shown in parentheses. (6) The variable of year was included in the models as an indicator variable. (7) *p < 0.1, **p < 0.05, and ***p < 0.01.

### Robust tests

In order test the robustness of the analysis at community level using datasets with missed information from a couple of communities, we further employed the data pooled at the county level for analysis. Table 4 reports the results of the robust tests. The results at county level (column 1–3) were found to be similar with the results at community level in terms of the signs, effect size, and the statistical significance.

**Table 4:** Robust test results.

Variables	County level		
	(1)	(2)	(3)
Number of healthcare workers per 1000 residents	− 1.723* (1.338)		− 3.791** (1.384)
Subsidy per staff		− 2.468*** (0.525)	− 2.788*** (0.547)
GDP per capita	5.579*** (1.602)	5.486*** (1.597)	5.458*** (1.458)
Year	Yes	Yes	Yes
*ρ*	0.249*** (0.055)	0.232*** (0.055)	0.233*** (0.055)
*N*	549	549	549

Note: (1) Number of healthcare workers, subsidy per staff, GDP per capita, and population are natural log transformed in regression analysis. (2) The unit of subsidy per staff is 1000 Yuan. (3) *ρ* represents spatial autocorrelation coefficient. (4) Standard errors are shown in parentheses. (5) The variable of year was included in the models as an indicator variable. (6) *p < 0.1, **p < 0.05, and ***p < 0.01.

The results of the distribution and LISA significance and cluster map of infectious diarrhea morbidity at county-level are encompassed in the Appendix, shown in Fig. S1 to S3. It is noteworthy that the infectious diarrhea morbidity of communities within each county could have significant heterogeneity, i.e. a few communities have high morbidity. The community-level analysis conducted in our study was capable of dealing with the heterogeneity issue which couldn’t be solved by county-level analysis conducted in previous studies.

## Discussions

In this study, the number of healthcare workers per 1000 residents was adopted as an indicator reflective of healthcare resource allocations among different primary healthcare institutions, which serves as an essential determinant of healthcare institutions’ performances in providing health services at the primary healthcare level. Moran’s I and its corresponding graphic tools were used to detect as well as visualize the global and local spatial autocorrelation of annual infectious diarrhea morbidities among different communities, based on which areas where increased healthcare manpower resources could be allocated were identified. The spatial lag fixed effects panel data model was then employed to explore the relationship between the number of primary healthcare workers per 1000 residents and the morbidity of infectious diarrhea at the community level. The Moran’s I analysis revealed the positive global autocorrelation of infectious diarrhea morbidity and identified high–high clusters to be mainly concentrated in regions with relative fewer amounts of primary healthcare workers. As indicated by the regression outcomes, a negative relationship was identified between the number of primary healthcare workers per 1000 residents and the morbidity of infectious diarrhea, with a 0.172 reduction of infectious diarrhea morbidity (1/10,000) associated with doubled amounts of primary healthcare workers per 1000 residents.

Similar with previous studies^16,44–46^, a positive correlation in the values of infectious diarrhea morbidities was found between different regions, indicating that the distribution of infectious diarrhea cases across Sichuan Province demonstrated obvious spatial clusters instead of being random. Through LISA analysis, significantly high–high and low–low clusters were found to be mainly distributed in western and eastern Sichuan during the studied period, which demonstrated consistency with regional economic development status as well as primary healthcare manpower resource allocations among different regions. It should be noted that these findings might have been induced by various factors such as regional economic development status, the penetration of WASH facilities, meteorological factors as well as primary healthcare resource allocations among different regions, thus should not be considered as evidences potent enough for indicating the direct association between the number of primary healthcare workers and infectious diarrhea morbidity at community levels. However, based on our findings as shown in Fig. 3, with an increased amount of high–high clusters emerging during the studied period, the prevention and control of infectious diarrhea cases in western Sichuan remains a critical issue that should be constantly addressed at both governmental and health administrative levels. It’s worth noting that most of low–high were near high–high clusters, indicating that in regions with high epidemics, some communities still demonstrated good work in the prevention and control of infectious diseases and had low infectious diarrhea morbidity.

The Spatial autocorrelation analysis suggested that a spatial statistical model instead of the classical linear regression should be adopted as a more appropriate method for exploring the relationship between the number of primary healthcare workers per 1000 residents and infectious diarrhea morbidities at the ecological level^47^. As the result, a spatial lag fixed effects panel data model was adopted in this study which was capable of taking both spatial autocorrelation and individual effects into consideration thus leading to higher statistical efficiency^47,48^. Specifically, biased outcomes produced by unmeasured potential confounders would be reduced by the adoption of spatial individual effects while the spatial correlation would be taken into account via the incorporation of a spatial weight matrix^49^.

Based on the regression results, a negative relationship was found between the number of primary healthcare workers per 1000 residents and infectious diarrhea morbidity at community levels, with a 0.172 reduction of infectious diarrhea morbidity (1/10,000) associated with doubled amounts of primary healthcare workers per 1000 residents. Our findings highlighted the pivotal role of primary healthcare workers allocation in the process of nationwide infectious diseases prevention and control in China. As previously mentioned in the Background section, the lack of primary healthcare workers in China has posed huge obstacles for primary healthcare institutions in achieving desired quality and efficiency of health service delivery in the process of infectious disease prevention and control. As the result, it is not difficult to predict that an increased amount of healthcare providers at primary healthcare level would significantly contribute to the reduction of nationwide infectious diarrhea morbidity via substantially improving the quality and efficiency of primary healthcare delivery. On the one hand, an increased amount of primary healthcare workers would made it more feasible for primary healthcare institutions to improve the whole team’s productivity and efficiency via assigning different tasks to particular staffs such as mosquitoes and flies control, sterilization, and disinfection. All kinds of tasks related to infectious disease prevention and control are more likely to be accomplished in a more efficient manner under more clearly defined jobs and responsibilities for different primary healthcare workers. On the other hand, it is not difficult to imagine that staffs assigned to specific tasks in a long-term are more likely to accomplish those tasks with improved quality and efficiency as they have obtained better knowledge through rich work experiences in those particular fields than general primary healthcare workers. Besides, from a general perspective, an increased amount of primary healthcare workers would substantially reduce the workload imposed on each individual staff thus improving the productivity and efficiency of the whole team. As previously presumed, a negative relationship was identified between subsidy per staff and infectious diarrhea morbidity, for which a reasonable explanation is that reduced subsidy is significantly associated with decreased motivation and productivity thus leading to higher infectious diarrhea morbidity as the result of poor disease prevention and control.

Through respectively comparing Fig. 1 with Fig. S1, Fig. 2 with Fig. S2 and Fig. 3 with Fig. S3, it can be concluded that significant heterogeneity might be embedded in community-level infectious diarrhea morbidities within each county. While infectious diarrhea morbidity was found to be low in most of the communities, a couple of communities demonstrated high infectious diarrhea morbidities. It is beyond controversy that the county-level analysis conducted in previous studies were not capable of identifying this issue thus leading to biased outcomes. However, it should be noted that biased outcomes would also be induced by community-level analysis due to increased amount of missing data if those data were not missing completely at random. In attempt to find an optimum solution for dealing with both heterogeneity and missing data issues, results produced at community levels were considered as the referential outcomes while county-level outcomes were used for robust analysis. Similar results were produced by these two kinds of analysis in terms of the signs, effect size, and the statistical significance, which indicated the rationality of our study.

Several limitations should be noted in this study. First, the study established a correlation rather than causality. As the result, biased regression outcomes might have been induced by endogenous problems potentially embedded in the established associations. For example, a reversed causality might exist between infectious diarrhea morbidity and the number of primary healthcare workers per 1000 residents. Specifically, an increased amount of primary healthcare workers might be allocated in areas with higher incidences of infectious diarrhea after disease outbreaks by governments, thus leading to underestimated impacts of primary healthcare workforce on infectious diarrhea morbidity. Second, as the sociodemographic variables cannot be obtained at the community level, our regression model only controlled for county GDP per capita instead. Although these factors would typically change very slowly across years and fixed effect has been handled in the regression model, the outcomes could still have been biased to some extent. Third, the infectious diarrhea cases could be underreported in underdeveloped regions, which could not be examined based on our dataset and would lead our regression estimates to be biased. Nevertheless, we do not think that the potential bias would make a big difference to the conclusions derived from the results of regression models as the actual higher infectious diarrhea morbidity would be in the underdeveloped regions, which would result in the sign of the regression estimates unaffected and the absolute values of the estimates underestimated.

## Conclusions

In summary, our study revealed the positive global autocorrelation of infectious diarrhea morbidity and identified high–high clusters to be mainly concentrated in regions with relative fewer amounts of primary healthcare workers. Besides, a negative relationship was also identified between the number of primary healthcare workers per 1000 residents and the morbidity of infectious diarrhea, with a 0.172 reduction of infectious diarrhea morbidity (1/10,000) associated with doubled amounts of primary healthcare workers per 1000 residents. Such findings were expected to provide evidence-based implications for policy-makers in the formulation of region-specific policies and strategies aimed at improving infectious diseases prevention and control in China.

## Methods

### Study area

Our study was based on Sichuan Province, a southwestern province in China, covering 183 counties and 4687 communities in the geographic regions of 97° 21′ to 108° 33′ east longitude and 26° 03′ to 34° 19′ north latitude. The land area and GDP per capita of Sichuan Province respectively ranked fifth and nineteen among 31 provinces of Mainland China, with a population of 83.41 million reported in 2018^21^. The socioeconomic and topographical characteristics vary significantly across the province, where eastern Sichuan is characterized by plains, dense population, and high-level economic development, while western Sichuan is in the opposite situation^50^. The resources of primary health care shows the same variation with the economic development in Sichuan^51^.

### Data sources

**Figure 4:**
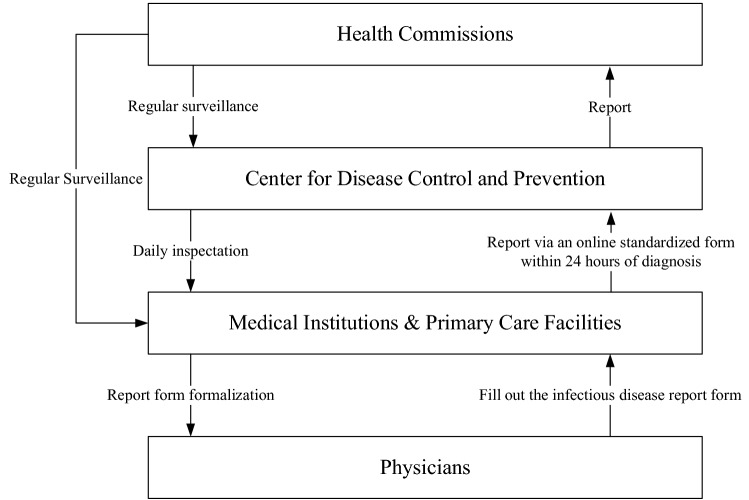
Infectious diarrhea cases surveillance and information feedback loop. The information is from Management of infectious disease information report, 2015 edition (http://www.gov.cn/xinwen/2015-11/11/content_2964135.htm).

Data retrieved from Sichuan Center for Disease Control and Prevention provided detailed information on both county-level and community-level yearly infectious diarrhea cases between January 1, 2017 and December 31, 2019. According to National Health and Family Planning Commission^52^, diarrhea is defined as a group of infectious diseases caused by various pathogens including bacteria, viruses, and parasites, with diarrhea as the typical symptom. In our study, diarrhea cases induced by dysentery, cholera, paratyphoid, and typhoid were not included due to data inaccessibility. As a notifiable infectious disease in China, all diarrhea cases should be reported via an online standardized form within 24 h of diagnosis^53^. The infectious diarrhea cases surveillance and information feedback loop is presented in the Fig. 4. More details of this loop can be found in this policy document^53^.

Data related to primary healthcare institutions from 2017 to 2019 were administrative data extracted from annual reports published by primary healthcare institutions and provided by Health Commission of Sichuan Province, which included the number of health workers and subsidy per staff. The number of health workers is calculated as the sum of the number of physicians, nurses, pharmacists, and clinical laboratory technicians. The subsidy per staff was adjusted for inflation rates, and was measured in 2019 RMB. We pooled these values at community-level according to the unique community code.

Information on the county GDP per capita was obtained from Sichuan Statistical Yearbook. The number of population at community-level in 2016 was obtained from Bureau of Statistics of Sichuan Province. We further inferred the values from 2017 to 2019 through multiplying the population weights (obtained from the number of population at community-level in 2016 divided by corresponding county-level population in 2016) by the number of population at county-level between 2017 and 2019.

We then used the unique community code to match the three kinds of sources of data. However, 366 communities were missed due to the different statistical gauge, accounting for 7.8% of total communities.

This study was approved by the Ethics Committee of Sichuan Center for Disease Control and Prevention. All methods were carried out in accordance with relevant guidelines and regulations. No confidential information was included because analyses were performed at the aggregate level. All data employed in this study were deidentified prior to analysis and anonymized confidentiality, thus informed consent was not required.

### Statistical analysis

#### Spatial autocorrelation analysis method

To capture the spatial distribution of infectious diarrhea morbidity and thus provide evidence-based implications for optimizing primary healthcare manpower resource allocations, Moran’s I and corresponding graphic tools are used to detect and visualize the global and local spatial autocorrelation of the annual infectious diarrhea morbidity^54^. In this study, we employed the row standardized first-order contiguity Rook neighbors as the criterion to identify neighbors. Given the rule, if regions *i* and *j* are adjacent, the spatial matrix w_*ij*_ = 1, otherwise, w_*ij*_ = 0. The global Moran’s I, ranging from 1 to − 1, was used to the overall spatial autocorrelation of all communities in Sichuan Province. Positive spatial autocorrelation values indicate regions with similar (low–low or high–high) infectious diarrhea morbidity are clustered together, while negative values indicate the opposite (cluster of dissimilar morbidity, low–high and high–low)^55^. Monte Carlo randomization (9999 permutations) was used to assess the statistical significance of Moran’s I, with the null hypothesis being that the distribution of infectious diarrhea morbidity in Sichuan province is completely random distributed^56^. Subsequently, local indicator of spatial association (LISA, local Moran’s I) and Moran scatterplots were used to detect the spatial autocorrelation of each communities in Sichuan Province and to identify the clusters’ location^57^. Statistically significant spatial clusters (high–low, region with high morbidity surrounded by region with low morbidity; and vice versa for low–low, low–high, high–high) were visualized using univariate LISA cluster map with community boundaries.

#### Spatial panel analysis method

In the study, annual infectious diarrhea morbidity and annual primary care facilities’ data pooled at community-level were used to explore the relationship between the number of primary healthcare workers per 1000 residents and the morbidity of infectious diarrhea. Because infectious diarrhea is a kind of infectious disease, the spatial heterogeneity and spatial dependence of the morbidity in different communities need to be captured in the analysis model^58,59^. A spatial panel data model is able to deal with the spatial heterogeneity and spatial dependence simultaneously, which is typically used to fit data containing repeated observations in different spatial units^47,48,60^. Spatial panel data possesses significant advantages compared to traditional cross-sectional or time series models, i.e. containing more variation and less collinearity among variables^47,61^, thus leading to the increase in estimation efficiency^47,49^. Given the results of model specification (shown in Table 2), the spatial lag fixed effects panel data model was ultimately employed to explore the relationship between the number of primary healthcare workers per 1000 residents and the morbidity of infectious diarrhea at community-level. The formula was set as follows:

1 $$Y_{it} = \rho \sum\limits_{j = 1}^{N} {w_{ij} Y_{jt} + \beta H_{it} + \gamma^{T} S_{it} + u_{i} + \eta_{t} + \varepsilon_{it} ,\quad i = 1, \ldots ,N,\;\;\;t = 1 \ldots ,T}$$

where *Y*_*it*_ denotes the infectious diarrhea morbidity for community *i* at year *t*. The parameter *ρ* is the spatial autoregression coefficient, indicating the impact degree of spatial factors on the dependent variable. *W*_*ij*_ represents the element of a (*N* × *N*) spatial weighting matrix. As previously mentioned, row standardized first-order contiguity Rook neighbours was employed to define the spatial weighting matrix. *H*_*it*_ indicates the number of primary care health workers per 1000 residents, which was pooled at community-level. *S*_*it*_ is the covariates representing the subsidy per staff and GDP per capita. The former variable is defined as the subsidy from governmental finance, superior departments, and other sources, divided by the number of staff in each primary care facility. To a certain degree, these subsidies can be used independently, and are typically used to purchase equipment, carry out public health programs, and pay staffs for bonus et al. This variable could be tightly related to the performance of primary healthcare workers in the prevention and control of infectious diarrhea, thus we included it in the model to better control for the potential confounding effect. The term *u*_*i*_ is the community specific effect and *η*_*t*_ represents time specific effect. *ε*_*it*_ is the error term and is assumed to be i.i.d. *N*(0, *σ*^2^) distribution.

All analyses were conducted using ArcGis 10.2 and R 3.6.3. *p* < 0.05 is used to determine statistical significance.

### Ethics approval and consent to participate

This study was approved by the Ethics Committee of Sichuan Center for Disease Control and Prevention. All methods were carried out in accordance with relevant guidelines and regulations. No confidential information was included because analyses were performed at the aggregate level. All data employed in this study were deidentified prior to analysis and anonymized confidentiality, thus informed consent was not required.

## Supplementary Information

Supplementary Information.

## Data Availability

The data that support the findings of this study are available from the Center for Disease Control and Prevention and Health Commission of Sichuan Province, but restrictions apply to the availability of these data. Data are can be made available from the authors upon reasonable request and with permission of the Center for Disease Control and Prevention and Health Commission of Sichuan Province.

## References

[1] Roth GA (2018). Global, regional, and national age-sex-specific mortality for 282 causes of death in 195 countries and territories, 1980–2017: A systematic analysis for the Global Burden of Disease Study 2017. Lancet.

[2] World Health Organization. The treatment of diarrhoea. http://whqlibdoc.who.int/publications/2005/9241593180.pdf (2005).

[3] Fischer Walker CL, Perin J, Aryee MJ, Boschi-Pinto C, Black RE (2012). Diarrhea incidence in low- and middle-income countries in 1990 and 2010: A systematic review. BMC Public Health.

[4] Lamberti LM, Fischer Walker CL, Black RE (2012). Systematic review of diarrhea duration and severity in children and adults in low- and middle-income countries. BMC Public Health.

[5] Dong S (2019). Morbidity analysis of the notifiable infectious diseases in China, 2018. CCDCW.

[6] Sarkar R (2014). Burden of diarrhea, hospitalization and mortality due to cryptosporidial infections in Indian children. PLoS Negl. Trop. Dis..

[7] Eisenberg JNS, Trostle J, Sorensen RJD, Shields KF (2012). Toward a systems approach to enteric pathogen transmission: From individual independence to community interdependence. Annu. Rev. Public Health.

[8] Walker CLF (2013). Global burden of childhood pneumonia and diarrhoea. Lancet.

[9] Baker KK (2018). Fecal fingerprints of enteric pathogen contamination in public environments of Kisumu, Kenya, associated with human sanitation conditions and domestic animals. Environ. Sci. Technol..

[10] Bozkurt A (2003). Association between household conditions and diarrheal diseases among children in Turkey: A cohort study. Pediatr. Int..

[11] Bbaale E (2011). Determinants of diarrhoea and acute respiratory infection among under-fives in Uganda. Australas. Med. J..

[12] Chowdhury M (2016). Low maternal education and socio-economic status were associated with household food insecurity in children under five with diarrhoea in Bangladesh. Acta Paediatr..

[13] Kumar S, Vollmer S, Kumar S, Vollmer S (2013). Does access to improved sanitation reduce childhood diarrhea in rural India?. Health Econ..

[14] Chard A (2020). Environmental and spatial determinants of enteric pathogen infection in rural Lao People’s Democratic Republic: A cross-sectional study. PLoS Negl. Trop. Dis..

[15] Hashizume M (2007). Association between climate variability and hospital visits for non-cholera diarrhoea in Bangladesh: Effects and vulnerable groups. Int. J. Epidemiol..

[16] Wangdi K, Clements A, Wangdi K, Clements ACA (2017). Spatial and temporal patterns of diarrhoea in Bhutan 2003–2013. BMC Infect. Dis..

[17] Aik J (2020). The effects of climate variability and seasonal influence on diarrhoeal disease in the tropical city-state of Singapore—A time-series analysis. Int. J. Hyg. Environ. Health.

[18] Hubbard S (2020). Household illness and associated water and sanitation factors in peri-urban Lusaka, Zambia, 2016–2017. NPJ Clean Water.

[19] Cairncross S (2010). Water, sanitation and hygiene for the prevention of diarrhoea. Int. J. Epidemiol..

[20] Reiner R (2020). Mapping geographical inequalities in childhood diarrhoeal morbidity and mortality in low-income and middle-income countries, 2000–17: Analysis for the Global Burden of Disease Study 2017. Lancet.

[21] National Bureau of Statistics of China. 2018 National statistical yearbook. http://www.stats.gov.cn/tjsj/ndsj/2019/indexch.htm (2019) **(in Chinese)**.

[22] Carlton E (2012). Regional disparities in the burden of disease attributable to unsafe water and poor sanitation in China. Bull. World Health Organ..

[23] Qu J, Fan M (2010). The current state of water quality and technology development for water pollution control in China. Crit. Rev. Environ. Sci. Technol..

[24] Zhang R (2009). Current situation analysis on China’s rural drinking water quality. Huan Jing Yu Jian Kang Za Zhi.

[25] Lamichhane P (2018). Does safe disposal of child faeces matter? An assessment of access to improved sanitation and child faeces disposal behaviour and diarrhoea in rural Nepal. Int. Health.

[26] Luby SP (2004). Effect of intensive handwashing promotion on childhood diarrhea in high-risk communities in Pakistan: A randomized controlled trial. JAMA.

[27] Chowdhury AMR, Chowdhury S, Islam MN, Islam A, Vaughan JP (1997). Control of tuberculosis by community health workers in Bangladesh. Lancet.

[28] Luby S (2005). Effect of handwashing on child health: A randomised controlled trial. Lancet.

[29] Olson CK (2011). Community case management of childhood diarrhea in a setting with declining use of oral rehydration therapy: Findings from cross-sectional studies among primary household caregivers, Kenya, 2007. Am. J. Trop. Med. Hyg..

[30] Olayo R, Wafula C, Aseyo E, Loum C, Kaseje D (2014). A quasi-experimental assessment of the effectiveness of the Community Health Strategy on health outcomes in Kenya. BMC Health Serv. Res..

[31] Chen T, Pan J (2020). The effect of spatial access to primary care on potentially avoidable hospitalizations of the elderly: Evidence from Chishui City, China. Soc. Indic. Res..

[32] Zhao, X., Zhang, Y., Yang, Y. & Pan, J. Diabetes-related avoidable hospitalisations and its relationship with primary healthcare resourcing in China: A cross-sectional study from Sichuan Province. *Health Soc. Care Community* n/a. 10.1111/hsc.13522 34309097

[33] Yi, P. Study on the perfect degree of infectious diseases prevention and control management and monitoring mechanism in Yunnan Province. Master’s thesis. Chongqing Medical University. https://kns.cnki.net/kcms/detail/detail.aspx?dbcode=CMFD&dbname=CMFDTEMP&filename=1020765047.nh&v=V6C8Bm6Gon3DHX8NDZP%25mmd2FrpuNo97ReNf7RzeCNwZ1WIQA6PoA4u5WPLmFDVTvOT%25mmd2FT (2020) **(in Chinese)**.

[34] Xu, Z. Dynamic modeling of infectious diseases in complex social systems and case studies. Doctor’s dissertation. Academy of Military Medical Sciences, accessed 13 October 2020; https://kns.cnki.net/kcms/detail/detail.aspx?dbcode=CDFD&dbname=CDFDLAST2015&filename=1015382235.nh&v=JgNo1C68QHhq3SzCW1AbMM8rOMdtQfVFj6ZSLuiZhmAJ4C%25mmd2BM5%25mmd2BrWxB1fwL3nN%25mmd2F%25mmd2BT (2015) **(in Chinese)**.

[35] National Health Commission. Law of the People’s Republic of China on the Prevention and Treatment of Infectious Diseases, accessed 13 October 2020; http://www.nhc.gov.cn/wjw/yjzj/202010/330ecbd72c3940408c3e5a49e8651343.shtml (2020) **(in Chinese)**.

[36] Wang, J. Research on multi-sectoral cooperation mechanism for infectious disease prevention and control in border areas of Yunnan province-a case study of the malaria control project in Pu’er City. Master’s thesis. Yunnan University of Finance and Economics, accessed 13 October 2020; https://kns.cnki.net/kcms/detail/detail.aspx?dbcode=CMFD&dbname=CMFD201602&filename=1016040428.nh&v=bHZt4dydzVqyxmECsQPj5I2Rx8FLr2QVCFfNGvIQWiruu4fisYn2tA9D675ZMcqv (2015) **(in Chinese)**.

[37] Health Commission of Sichuan Province. 2019 statistical bulletin of the health development of Sichuan Province, accessed 13 October 2020; http://wsjkw.sc.gov.cn/scwsjkw/njgb/2020/3/31/6922a434a6454fc481f037bbaa9031da.shtml (2020) **(in Chinese)**.

[38] Liao C, Yu D (2018). Investigation and analysis of health education and health promotion in Bazhou District. Healthmust-Readmagazine..

[39] Chen Y (2012). Investigation on health education in primary care facilities in Wenjiang District, Chengdu. Chin. Health Ind..

[40] Zhou, Y. & Yu, M. The present situation and countermeasures of health education and health promotion in primary care facilities. *Psychologies* **2**, 168 (2018) **(in Chinese)**.

[41] Huang J, Yang M, Zhang J (2020). Analysis of risk factors of blood pressure control in rural hypertension patients in Sichuan Province. Modern Prev. Med..

[42] Zhang Y, Ming J, Zhu J, Zheng X, Hu J (2012). A study on the current situation of public health service in township health centers at different economic levels regions in Sichuan Province. Chin. Health Serv. Manag..

[43] Fu D (2020). Research on the status quo of health education and health promotion in primary care facilities. China Health Care Nutr..

[44] Faria C (2017). Geospatial distribution of intestinal parasitic infections in Rio de Janeiro (Brazil) and its association with social determinants. PLoS Negl. Trop. Dis..

[45] Ma Y (2015). Spatio-temporal pattern and socio-economic factors of bacillary dysentery at county level in Sichuan Province, China. Sci. Rep..

[46] Tampah-Naah A (2019). Geospatial analysis of childhood morbidity in Ghana. PLoS ONE.

[47] Barufi AM, Haddad E, Paez A (2012). Infant mortality in Brazil, 1980–2000: A spatial panel data analysis. BMC Public Health.

[48] Wang H (2015). Detecting the association between meteorological factors and hand, foot, and mouth disease using spatial panel data models. Int. J. Infect. Dis..

[49] Ponicki WR, Gruenewald PJ, Remer LG (2013). Spatial panel analyses of alcohol outlets and motor vehicle crashes in California: 1999–2008. Accid. Anal. Prev..

[50] Liu M, Qin X, Pan J (2017). Does medical equipment expansion lead to more diagnostic services? Evidence from China’s Sichuan Province. Emerg. Mark. Financ. Trade.

[51] Wang X, Yang H, Duan Z, Pan J (2018). Spatial accessibility of primary health care in China: A case study in Sichuan Province. Soc. Sci. Med..

[52] National Health and Family Planning Commission. Diagnostic criteria and principles of management of infectious diarrhea (WS 271-2007). http://www.gxcdc.com/uploadfile/2017/0515/20170515043033854.pdf (2007) **(in Chinese).**

[53] National Health and Family Planning Commission. Management of infectious disease information report, 2015 edition. http://www.chinacdc.cn/jkzt/crb/xcrxjb/201810/t20181017_195160.html (2015) **(in Chinese)**.

[54] Fischer MM, Griffith DA (2008). Modeling spatial autocorrelation in spatial interaction data: An application to patent citation data in the European Union*. J. Reg. Sci..

[55] Xia J (2015). Spatial, temporal, and spatiotemporal analysis of malaria in Hubei Province, China from 2004–2011. Malar. J..

[56] Wu W, Guo J, Guan P, Sun Y, Zhou B (2011). Clusters of spatial, temporal, and space-time distribution of hemorrhagic fever with renal syndrome in Liaoning Province, Northeastern China. BMC Infect. Dis..

[57] Anselin L (1995). Local indicators of spatial association—LISA. Geogr. Anal..

[58] Liu Y (2012). Investigation of space-time clusters and geospatial hot spots for the occurrence of tuberculosis in Beijing. Int. J. Tuberc. Lung Dis..

[59] Wei Y (2014). Rapid increase of scrub typhus: An epidemiology and spatial-temporal cluster analysis in Guangzhou City, Southern China, 2006–2012. PLoS ONE.

[60] Ehlert A, Oberschachtsiek D (2014). Does managed care reduce health care expenditure? Evidence from spatial panel data. Int. J. Health Care Finance Econ..

[61] Kelejian HH, Prucha IR (2010). Specification and estimation of spatial autoregressive models with autoregressive and heteroskedastic disturbances. J. Econom..

