# Supplementary material for: Exploring the effect of the primary care health workers number on infectious diarrhea morbidity and where the health resources should go
Source: Sci Rep. 2022 Apr 11;12:6060. doi: 10.1038/s41598-022-10060-y (PMC9001693; doi:10.1038/s41598-022-10060-y)
Supplement: Supplementary file 1 — Supplementary Information. [file 41598_2022_10060_MOESM1_ESM.doc]

# Supplementary materials

# Article title

# Exploring the effect of the primary care health workers number on infectious diarrhea morbidity and where the health resources should go.

# Authors

# Xujing Guan†, Tianjiao Lan†, Weibin Liao , Xue’er Wu, Jay Pan

# Corresponding authors

Jay Pan, West China School of Public Health and West China Fourth Hospital, HEOA Group, Sichuan University and West China Research Center for Rural Health Development, Sichuan University. Address: West China School of Public Health, Sichuan University, Chengdu, China, 610041. Email: [panjie.jay@scu.edu.cn](mailto:panjie.jay@scu.edu.cn).

^†^ These authors contributed equally to this work

# List of Supplementary materials

[Figure S.1 The geographical distribution of infectious diarrhea morbidity at county-level from 2017 to 2019 3](#__RefHeading___Toc70517111)

[Figure S.2 Moran scatter plot for the annual infectious diarrhea morbidity at county-level from 2017 to 2019 4](#__RefHeading___Toc70517112)

[Figure S.3 LISA significance map and cluster map for infectious diarrhea morbidity at county-level in Sichuan Province, China, 2017-2019 5](#__RefHeading___Toc70517113)

# Figure S.1 The geographical distribution of infectious diarrhea morbidity at county-level from 2017 to 2019


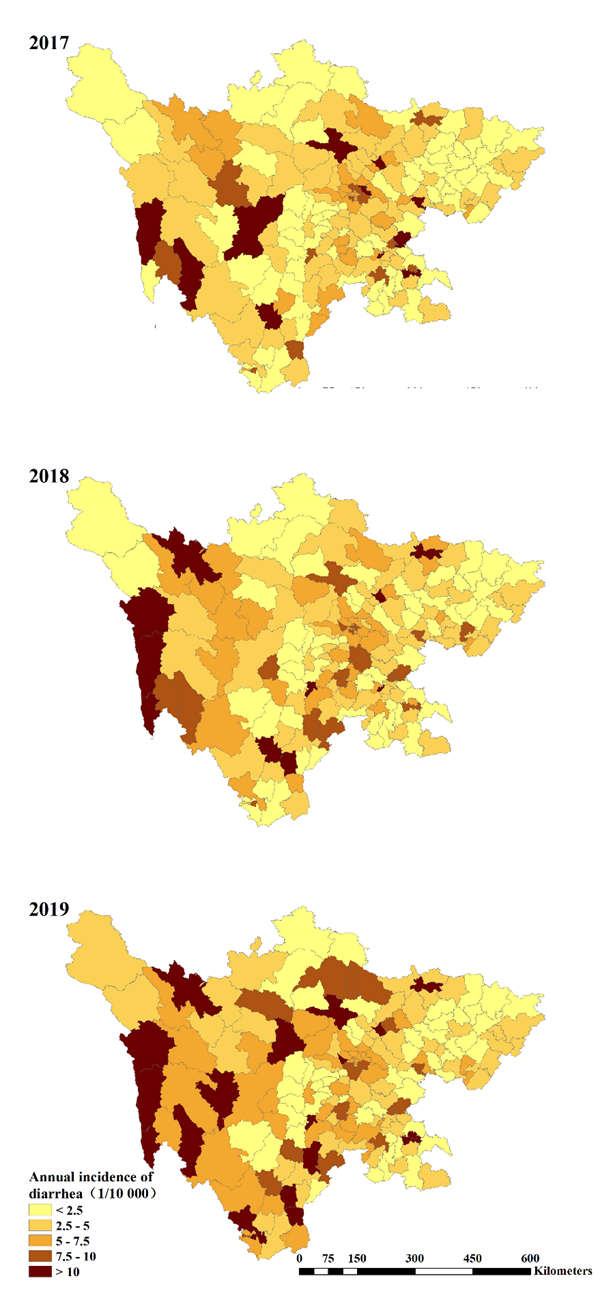


**Figure S.1** The geographical distribution of infectious diarrhea morbidity at county-level from 2017. The map was created by ArcGIS software (version 10.0, authorization number: EFL734321752, URL: https://developers.arcgis.com/)

# Figure S.2 Moran scatter plot for the annual infectious diarrhea morbidity at county-level from 2017 to 2019


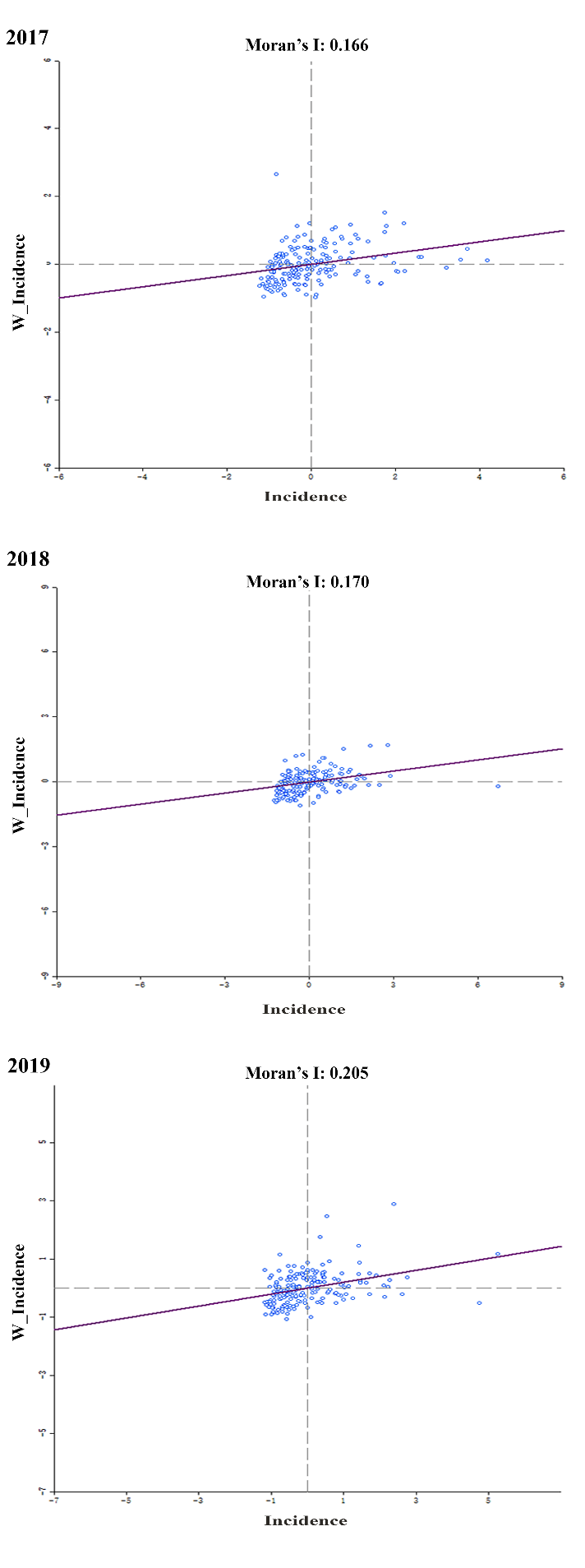


**Figure S.2** Moran scatter plot for the annual infectious diarrhea morbidity at county-level from 2017 to 2019

# Figure S.3 LISA significance map and cluster map for infectious diarrhea morbidity at county-level in Sichuan Province, China, 2017-2019


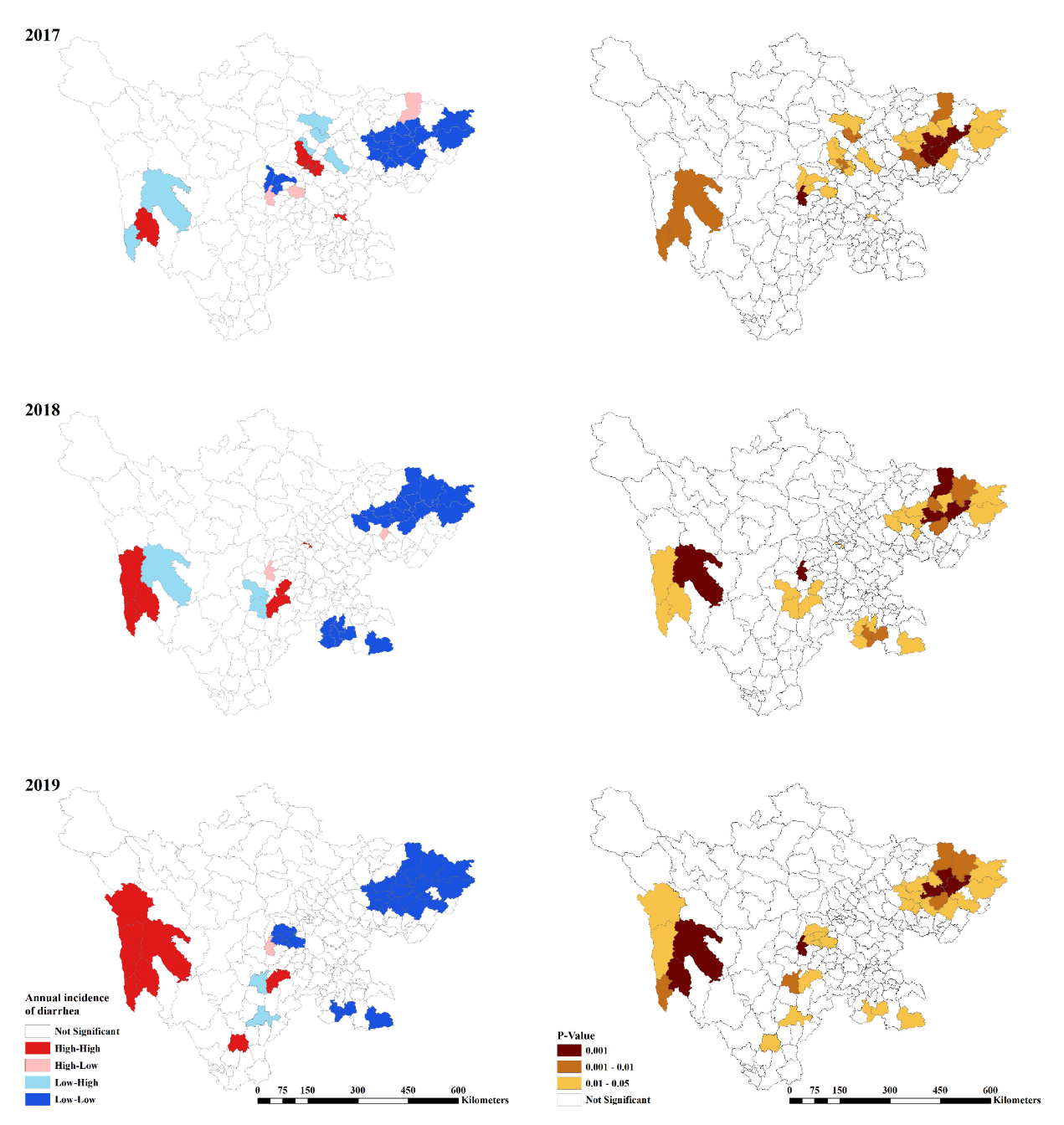


**Figure S.3** LISA significance map and cluster map for infectious diarrhea morbidity at county-level in Sichuan Province, China, 2017-2019. The map was created by ArcGIS software (version 10.0, authorization number: EFL734321752, URL: https://developers.arcgis.com/)
